# Educational and Exercise Intervention to Prevent Falls and Improve Participation in Subjects With Neurological Conditions: The NEUROFALL Randomized Controlled Trial

**DOI:** 10.3389/fneur.2019.00865

**Published:** 2019-09-13

**Authors:** Davide Cattaneo, Elisa Gervasoni, Elisabetta Pupillo, Elisa Bianchi, Irene Aprile, Isabella Imbimbo, Rita Russo, Arianna Cruciani, Andrea Turolla, Johanna Jonsdottir, Michela Agostini, Ettore Beghi, NEUROFALL Group, Angelo Montesano

**Affiliations:** Milano; Venezia.; ^1^Fondazione Don Carlo Gnocchi Onlus (IRCCS), Milan, Italy; ^2^Istituto Di Ricerche Farmacologiche Mario Negri, Milan, Italy; ^3^San Carlo Borromeo Hospital, Milan, Italy; ^4^IRCCS Fondazione Ospedale San Camillo, Venice, Italy

**Keywords:** prevention, falls, participation, neurological disease, rehabilitation

## Abstract

**Background:** Falls, mobility impairments and lack of social support lead to participation restrictions in people with neurological conditions. The aim of this multicenter, single blinded randomized controlled trial was to test whether an educational program focusing on fall prevention and safe mobility reduces falls and increases social participation among people with neurological conditions.

**Methods:** Ninety people with Stroke (*n* = 25), multiple sclerosis (*n* = 33) and Parkinson disease (*n* = 32), median age 63 (31–89), were randomized. A permuted block algorithm stratified by field center was used to allocate participants to an education group (EG, *n* = 42) consisting of an educational program focused on fall prevention and tailored balance exercises and a control group (CG, *n* = 48) receiving usual treatments. After baseline assessment, each participants was followed for 6 months with telephone contacts by blinded interviewers. Being fallers (>1 fall) and time to become a faller were used as primary outcomes. Community Integration Questionnaire (CIQ) and Instrumental Activities of Daily Living (IADL) scales assessed treatment effects on social integration and daily living activities.

**Results:** Over a median (Interquartile Range) follow-up of 189 (182–205) days, [EG = 188 (182–202), CG = 189 (182–209)] fallers were 10 in the CG and 11 in the EG (hazard ratio 0.95, 95% confidence interval (CI) 0.45 to 2.5; *P* = 0.94). At follow-up the EG scored significantly better than CG on the CIQ (+1.7 points, CI: 0.1 to 3.3) and IADL (+2.2 points, CI: 0.4 to 4.0).

**Conclusions:** This educational program did not reduce the risk of falls but it improved the ability to carry out activities of daily living and decreased participation restrictions in people with neurological conditions.

## Introduction

People with neurological disorders are often characterized by motor dysfunction and imbalance leading to risk of falls and impacting on participation in activities of daily living.

Parkinson disease (PD), multiple sclerosis (MS) and stroke carry high risk of falls. Among people with PD, 45–68% are reported to be fallers each year and two-thirds of them fall repeatedly (1). More than 50% of people with MS are fallers (2) and about 14–65% of participants with stroke fall at least once while in hospital and 37–73% fall during the 6 months after discharge (3).

In a recent observational study, falls, mobility impairments and lack of social support led to participation restrictions in 77% of participants with MS (4). This is in keeping with other studies showing lower level of participation in PD (5) and stroke (6) and difficulties in the execution of home, social and productive activities. Although participation has recently been suggested as a primary outcome of interventions (7), little is known of the effects of rehabilitation on participation restrictions and the relationship between participation in social activities and falls.

A published report investigating the risk of falls and fall predictors in 299 people with PD, MS, and stroke (8) showed that 47.1% of participants fell at least once in the 6 months following baseline assessment and 31.7 and 17.0% of the sample reported repeated and, respectively, injurious falls.

These results are in line with previous studies showing high proportion of fallers among these three conditions often leading to injuries and impairments in activities of daily living (1, 9, 10). A second study inquired on pathology-specific mobility and balance disorders associated with falls and participation restrictions (11). However, there is insufficient evidence on the effects of rehabilitation on fall prevention (12) and even less on the effects of a combined educational and exercise program to reduce fall frequency (13).

Here we report results associated with the hypothesis that a combined educational and exercise program focusing on fall prevention and safe mobility reduces the risk of falls and increases social participation among subjects with PD, MS, and stroke with functional limitations.

## Methods

Our study was a multicenter, single blinded randomized trial conducted in three Italian field centers between January 2015 and March 2016 by the NEUROFALL group. This group comprised researchers and clinicians (physiotherapists and medical doctors) involved in studies on fall prediction and prevention in neurological disorders.

Participants were included if they had PD, MS or stroke, were able to walk 10 m independently with or without a mobility aid, were willing to commit to the educational program, and were able to give written informed consent. A patient was excluded if he/she had: (1) Major depression; (2) Severe joint/bone disorder interfering with mobility; (3) Aphasia if interfering with understanding the aims of the study and self-administered tests; (4) relapses in the previous 3 months (MS); (5) Stroke occurred in <4 weeks before study entry; (6) Cognitive impairment (Minimental State Examination score <21); To increase generalizability of findings we did not exclude subjects with a MMSE lower than 24. However, we asked caregivers to interact with assessors to check for data consistency in participants with MMSE scores from 21 to 24. The institutional review boards at all participating sites approved the study protocol (FDG_10.12.2014), and written informed consent was obtained from all participants. The study was registered in ClinicalTrials.gov (NCT03570268).

### Randomization

**Figure 1:**
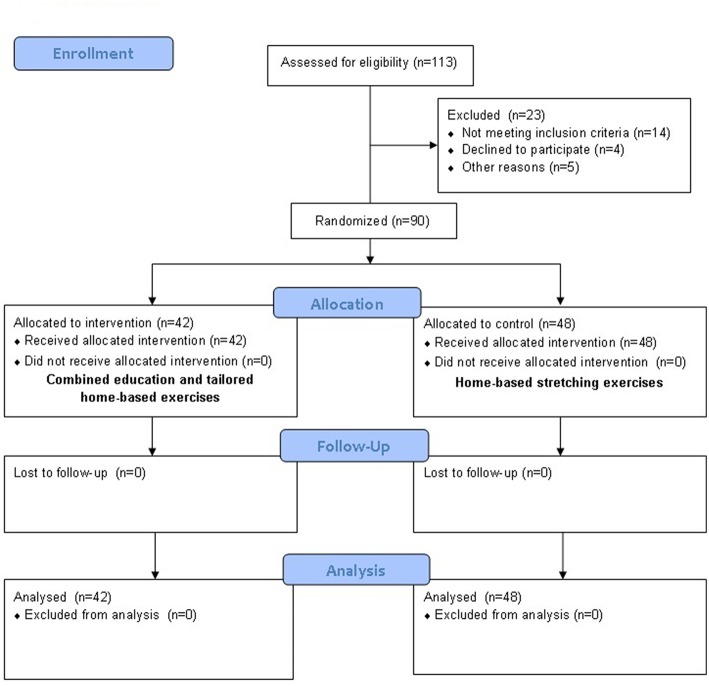
CONSORT flow chart.

Participants were randomized 1:1 to an education or control group (see Figure 1) using a computer generated randomization list generated before commencement of the study and stratified by field center and pathology. Randomization sequence was created with SAS 9.2 (SAS Institute, Cary, NC, USA) using random block sizes of 4.

### Interventions

Participants in the experimental intervention group (education group) received an educational program and tailored home exercises. From a theoretical point of view the educational intervention drew upon educational group theory, and the delivery of recently published papers investigating the effect of educational programs (14, 15).

From a practical point of view the educational program consisted of a mix of peer to peer and clinician led session, lasting 1 h. The session consisted of multiple, interacting components fostering brainstorming, problem-solving and action planning activities (16) and was supported by a handbook and audio-video material designed to promote sharing of strategies used to prevent falls and to improve social participation and activity of daily living among participants. We also provided videos of falls to foster discussion among participants on causes, circumstances and behaviors leading to falls.

The 1 h session was led by a trained physical therapist who delivered information to small groups ranging in size from two to four people with the same pathology. Regarding the contents, we focused on increasing knowledge of pathology-specific types of falls, behavioral and environmental fall risk factors such as internal and external barriers. Participants were asked to provide examples of falls reporting their feelings and the strategies they used to modify their behaviors and environment to reduce fall risk factors. We provided information on participation restrictions and its effects on eventually increasing deconditioning and falls and then asked the participants to comment on the trade-off between falls prevention (e.g., restricting activities of daily living when fatigue) and independence in activity of daily living. Finally, we asked participants to share their strategies to maintain an active lifestyle while reducing risk of falling. At the end of the session the therapist moderating the group provided information on techniques and strategies for preventing falls and increase social participation and engagement in activities of daily living that were not covered during the session.

After the educational session we provided two 1 h exercise sessions to teach the exercises and 1 h follow up session 2 days after the last exercise sessions.

The exercise sessions were spent to teach tailored mobility and balance exercises developed on existing evidence (17), our previous research (18–20) and clinical experience. In the follow-up session participants discussed issues from the preceding session, they were asked if they had understood the study procedure and the tasks they needed to carry out at home and were supervised while they performed the prescribed exercises in order to correct eventual errors.

Participants were invited to perform the exercises at home 2–3 times a week for 2 months.

Participants allocated to the control group received ongoing usual treatments. In addition, two 1 h sessions were spent to teach stretching exercises that the patient was invited to perform at home for 2 months.

Subjects in both groups were asked not to discuss the intervention with other participants to avoid patients in the usual care group integrating components of the intervention into their routine.

### Data Collection

After releasing written informed consent, each participant was invited to a baseline visit to collect demographic and clinical characteristics including age, sex, disease type and duration, use of walking aids, incontinence, and history of falls in the preceding 6 months. A number of clinical scales were used to test functional disability only at baseline, balance (Berg Balance Scale, BBS) (21), walking abilities (Timed Up & Go, TUG, Ten Meter Walking Test) (22). and self-confidence with balance (Activity Balance Confidence Scale, ABC) (23). Questionnaires on performance during daily living activities (Instrumental Activities of Daily Living, IADL) (24), and social integration (Community Integration Questionnaire, CIQ) (25) were applied at baseline and at follow up. Each patient was given a fall diary and was followed for 6 months with telephone contacts approximately at 2, 4, and 6 months. At each contact, the patient was inquired on targeted mobility and balance rehabilitation programs received during the follow up, use of walking aids, and any incurred falls, with date, circumstances, underlying cause and related injuries. A fall was defined as an unexpected event where the person inadvertently came to rest on the ground or other lower level (26). Subjects with >1 fall in the 6 months follow up were categorized as “fallers.” Percentage of fallers and time to second fall were used as primary outcomes.

CIQ and IADL scales were rated at last follow up to assess the effects of intervention on changes in social integration and daily living activities. Data were collected by trained interviewers blinded to the intervention not located in the clinical centers where the assessments were made. Data was uploaded in an *ad-hoc* database and was not available until the end of the study.

### Statistical Analysis

We assessed the impact of education on the cumulative time-dependent probability of falls with percentage of fallers as our primary outcome variable. A subject was categorized as a faller at the time of the second fall occurring after randomization. Event time was defined as the time from first assessment until participant was categorized as faller, and censoring times were defined as the time from first assessment until the fall assessment. Kaplan–Meier estimator was used to obtain cumulative incidence curves for the education and control group. For group comparison, we estimated hazard ratios with 95% confidence intervals and used likelihood ratio tests from Cox proportional hazards regression models. A multivariable analysis was also performed controlling for pathology, falls number at baseline, age, disease duration, treatment received during the FU and use of walking aid.

For the clinical outcomes, we focused on participation (CIQ) and activities of daily living (IADL). For each measure, we used multivariable linear models to compare the specific intervention effect on total scores, for subjects in the education and control group, adjusting for baseline score, pathology, falls number at baseline, age, disease duration, treatment received during the FU and use of walking aid. The covariates were selected as they represented main fall risk factors in participants with neurological conditions (2, 27, 28). The effect of intervention was assessed on a two-tailed significance of 0.05 using the intention to treat approach.

In a previous study conducted in a similar population, about 40% of patients experienced at least one fall over a 6 month period, and about 25% experienced at least two falls over the same period (25). Assuming that the percentage of patients experiencing at least one fall would decrease to 15% in the education group, we planned to include 48 patients per group to have 80% power to detect this difference with a 5% level of significance. With this planned sample size, the study also had sufficient power (80%) to detect a reduction of 5% in the percentage of patients experiencing at least two falls in the education group.

As only five (5.5%) patients were categorized as “injurious fallers” (i.e., fallers experiencing injuries) at the follow-up (3 education group, 2 control group) in this study we did not report results for this variable.

## Results

A total of 113 patients were assessed for eligibility (Figure 1). Ninety cases (MS = 33, PD = 32, Stroke = 25) were randomized to the education group (*n* = 42) or to the control group (*n* = 48). The planned number (48 patients in each arm) could not be achieved because the recruitment rate was increasingly slow, preventing the enrolment of the six additional patients within an acceptable time frame. With 42 patients enrolled in the experimental group, instead of the 48 originally planned, the power for the primary endpoint is reduced to 76%. The baseline characteristics of the two intervention groups were comparable (Table 1).

**Table 1:** Clinical and demographic characteristics of the sample.

	**Education group (*n* = 42)**	**Control group (*n* = 48)**
Variable	**Demographic characteristics**	
Age in years mean (SD)	61 (15)	63 (11)
Women *N* (%)	16 (38)	17 (35)
	**Clinical characteristics**	
Pathology		
Multiple sclerosis *N* (%)	16 (38)	17 (35)
Parkinson *N* (%)	15 (36)	17 (35)
Stroke *N* (%)	11 (26)	14 (29)
Incontinence *N* (%)	14 (34)	11 (25)
Disease duration in years mean (SD)	9 (7)	9 (7)
Falls in past six months median (IQR)	1 (0-3)	0 (0–2)
	**Walking aid**	
Walking Aid		
None *N* (%)	14 (34)	20 (42)
Unilateral *N* (%)	8 (19)	13 (27)
Bilateral *N* (%)	16 (38)	9 (19)
Wheelchair *N* (%)	10 (4)	6 (13)
	**Functional characteristics**	
BBS median (IQR)	42 (37–47)	42 (35–48)
TUG (seconds) median (IQR)	26 (26)	24 (25)
10m Walking Test (second) mean (SD)	20 (31)	17 (29)
IADL median (IQR)	14 (10–17)	13 (9–17)
ABC median (IQR)	45 (33–69)	48 (25–70)
CIQ median (IQR)	12 (9–15)	13 (10–15)

*BBS, Berg Balance Scale; TUG, Timed up and go Test; IADL, Instrumental Activity of Daily Living; ABC, Activities Balance Confidence; CIQ, Community Integration Questionnaire; SD, Standard Deviation; IQR, Interquartile range, (Q_1_-Q_3_)*.

Thirty participants (33%) reported more than 1 fall in the past 6 months and 71 (79%) received rehabilitation during the follow-up. Patients with history of falls were slightly more frequent in the experimental group (18.42%) compared to the control group (12.25%). However, no statistically significant between group differences were found for percentage number of fallers (Educatio*n* = 25.60%; Control = 12.25%; *p* = 0.12) and number of subjects receiving other treatments (Educatio*n* = 35.85%; Control = 36.75%, *p* = 0.48), (Supplementary Figure 1).

**Figure 2:**
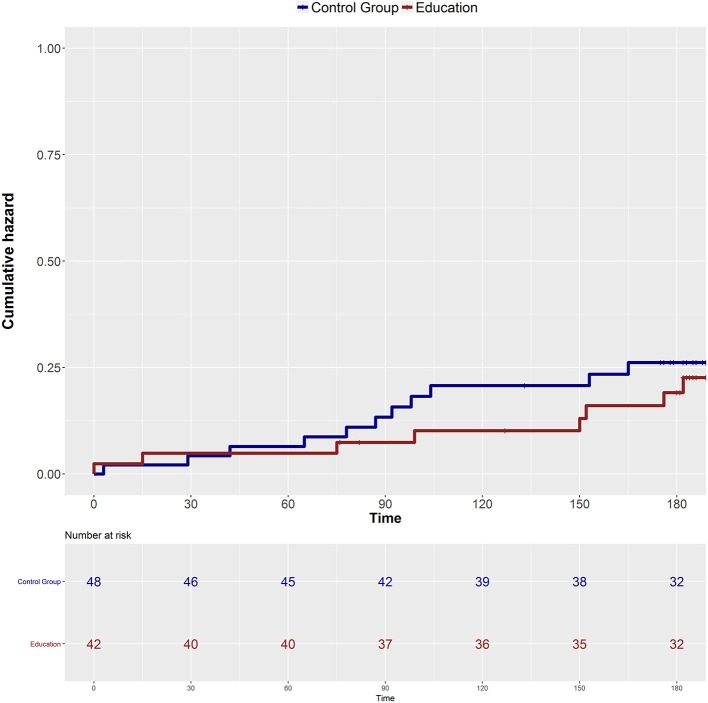
Cumulative hazard of falling over time in the two groups.

Over a median (IQR) follow-up of 189 (182–205) days, [Education = 188 (182–202), Control = 189 (182–209)] 10 participants (24%) in the education group were fallers (had fallen twice) and 11 (23%) in the control group were fallers (adjusted hazard ratio 0.95, 95% confidence interval (CI) 0.45 to 2.5; *P* = 0.94, Figure 2). The results were unchanged when the number of falls at baseline, age, disease duration, treatment received during the follow-up and use of walking aid were accounted for (adjusted hazard ratio 0.61, 95% CI 0.57 to 4.6; *P* = 0.35).

**Figure 3:**
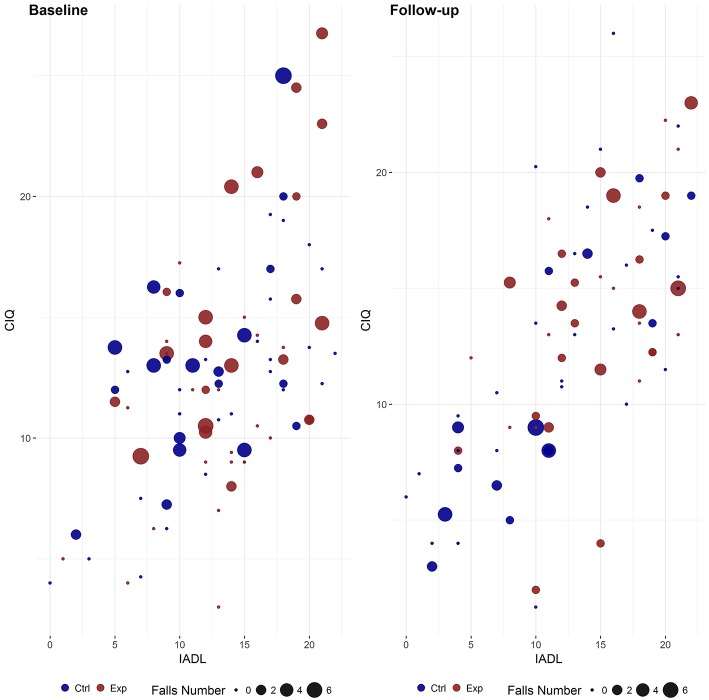
Relationship between participation, activity of daily living and number of falls in education and control group at baseline and during follow-up. IADL, Instrumental Activity of Daily Living; CIQ, Community Integration Questionnaire; Exp, Experimental Group; Ctrl, Control Group.

A clinically meaningful relationship was observed between number of falls, CIQ and IADL (Figure 3) suggesting that number of falls in the education group were evenly distributed at baseline, while post treatment a high number of falls was present only in subjects with higher level of participation and independence in activities of daily living.

Based on linear models, the education group averaged 1.7 (CI: 0.1 to 3.3) more points on the CIQ than the control group (*P* = 0.04). Subjects using walking aids scored lower than subjects walking without support.

**Figure 4:**
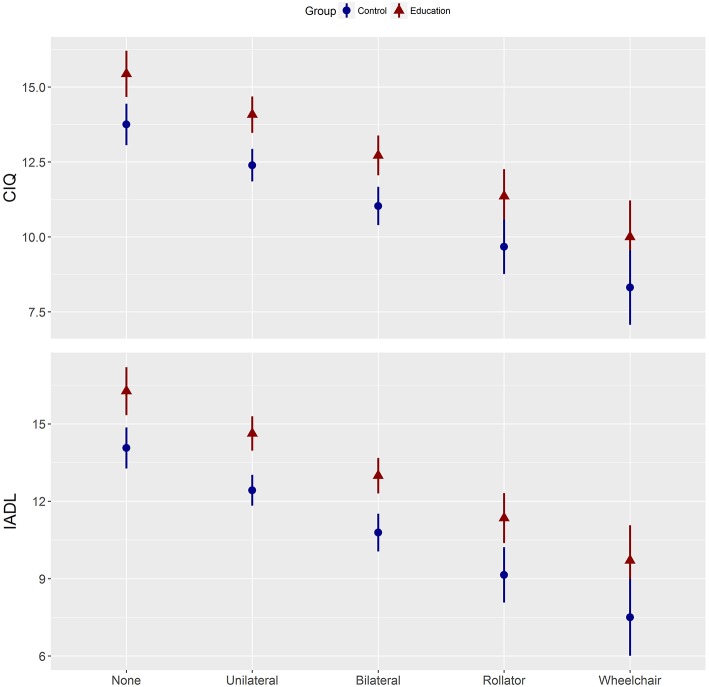
Participation and activity of daily living post-scores adjusted for pre-scores. CIQ, Community Integration Questionnaire; IADL, Instrumental Activities of Daily Living; Control, home-based stretching exercises group; Education, combined education and tailored home-based exercises (experimental) group.

Figure 4 reports CIQ post-scores adjusted for pre-scores, treatment received during follow-up, and walking aid for the education and control group. Similarly, the adjusted IADL scores during follow-up (Figure 4) were greater for the education group than for the control group with a mean between group difference of 2.2 (CI: 0.4 to 4.0, *P* = 0.02) and, with subjects walking without support showing best scores.

## Discussion

Several studies have investigated the effects of exercise on falls prevention with negative results (12, 13). However, few reports investigated the combination of multifactorial interventions (29). Our hypothesis was that the combination of educational sessions and home exercises could reduce falls without a concomitant reduction in activities daily living and participation. However, we found no difference in the risk of falls for an educational intervention vs. a usual care program among participants with neurological conditions with gait and balance limitations and high risks of falling. The results were consistent across several subgroups, including pathology and history of falls. On the other hand, the education program improved ability to carry out activities of daily living and decreased participation restrictions without a concomitant increase of number of falls.

Our sample was at high risk of falls as evidenced by 33% of participants reporting one or more falls in the 6 months before assessment. The participants had functional limitations, with a mean BBS score of 42 points at baseline that is below the expected score of 55 (SD 2.5) points for healthy people of similar age (30) and below the cut-off score of 45 points for the occurrence of balance disorders (31).

Despite this being a fairly large trial of education in subjects with neurological conditions we did not find a reduction in the proportion of fallers after the intervention. This is in keeping with previous studies showing that well-designed approaches comprising only exercises can enhance mobility in stroke (32, 33) but their effects on falls are still unclear (10). A recent review comprising seven studies reporting exercise intervention did not show a significant reduction in the rate of falls in the acute and subacute stages after stroke (12).

Mixed results were also reported for PD since the only two trials showing reductions in fall frequency (34, 35) were fully supervised. Fall prevention in MS also showed mixed results with some studies showing 22–35% reduction of fallers in the experimental groups while others reported no effects (14, 20).

There may be several explanations for the lack of effects of our combined education and exercise program on falls. Firstly, at endpoint people in the educational group showed higher levels of daily activities and participation than those in the control group. It is thus possible that the risk abatement was in part compensated by an increase in risk behavior in the education group.

Secondly, we used falls diaries to assess the number of falls in the 6 months after baseline assessment, after having explained to participants at recruitment how to complete the diaries. Unlike more sophisticated devices, the diaries are inexpensive and easy to complete. Nevertheless, it is possible that misreporting falls may have occurred differently in the two groups.

Furthermore, it was impossible for ethical and practical reasons to restrain rehabilitation treatments in our cohort for 6 months. These activities were monitored during the follow up period and almost 80% of the sample received treatment to improve balance and gait. Although between group differences in treatment received should be controlled by random allocation of subjects and were included as covariate in cox models, their effects may have biased the results of our intervention.

Nonetheless, the education improved scores on CIQ, a measure of home and social integration, and productive activities and on activities of daily living. This is of importance since reduction of falls after interventions might otherwise have been attributed to restricted participation in activities of daily living. Moreover, improvement in participation may ultimately prove to be beneficial by leading to functional improvement and decreasing falls in the long term.

The effects of rehabilitation on participation and activities of daily living are still unclear with some reviews and studies showing no effects (36) while others report improvements after occupational therapy and functional electrical stimulation (37). Our results corroborate findings from a recent review suggesting that self -management programs can improve quality of life in people with neurological conditions. Further studies are needed to understand if new more complex multidisciplinary, personalized, and patient-centered approaches with an efficient involvement of caregivers and family members can have stronger impact on participation in people with neurological conditions (38).

### Study Limitations

The first limitation is the selection of the study population since this is not a population-based study. Second, we selected prevalent rather than incident participants in need of rehabilitation. The exclusion of participants who were not proposed for rehabilitation programs might have resulted in a selected sample. Third, the collection of data on falls was dependent on the compliance of the participants and, although they were provided with fall diaries, the 2 month intervals between our assessments might have been too long to prevent recall bias. Fourth, the follow-up was perhaps too short for a precise detection of fallers. Fifth, although the total sample is large, the participants affected by each disease were perhaps too few for the detection of disease-specific differences in the rate of falls after intervention. Last, the planned number of patients to be randomized could not be achieved due to an increasingly low recruitment rate. However, given the 1% difference in the percentage of fallers between the two treatment arms, a statistically significant difference in favor of the educational arm could not be obtained even in the unlikely event that all cases had been randomized to the experimental arm and none of them became fallers during follow-up.

Even with these limitations, our study documents that the combined education program improved the ability to carry out activities of daily living, decreasing participation restrictions without a concomitant increase of number of falls. Further studies with a better methodology are needed to fully exploit the effect of the combination of educational and balance training program on fall reduction in the different neurological groups. First, according to Finlayson et al. (14) a delivered group program lasting several weeks may result in better outcomes. Moreover, the provision of more group activities, lectures and take-home exercises might better reinforce program contents. Periodical supervising of home exercises should be introduced to tailor treatments according to participant improvements, deliver better scheduling of treatment sessions, motivate subjects and provide feedback of performance. Finally, environmental assessments, prescription of appropriate mobility aids, and the involvement of the caregiver should be considered to reduce fall frequency.

## Ethics Statement

This study was carried out in accordance with, and the protocol approved by, the recommendations of Fondazione Don Gnocchi.

## Author Contributions

DC, EG, and EBe drafted the manuscript. EBi conducted the statistical analysis. IA, II, AC, RR, AT, MA, and the NEUROFALL Group collected the data. DC, EG, JJ, and EBe participated in project conception, organization and execution. All the authors reviewed and critiqued the manuscript.

### Conflict of Interest Statement

The authors declare that the research was conducted in the absence of any commercial or financial relationships that could be construed as a potential conflict of interest.

## Supplementary Material

Supplementary Figure 1 Hazard ratios for time to fall for education vs. control group according to subgroups.
