# Supplementary figures and images for: Educational and Exercise Intervention to Prevent Falls and Improve Participation in Subjects With Neurological Conditions: The NEUROFALL Randomized Controlled Trial
Source: Front Neurol. 2019 Sep 13;10:865. doi: 10.3389/fneur.2019.00865 (PMC6754067; doi:10.3389/fneur.2019.00865)

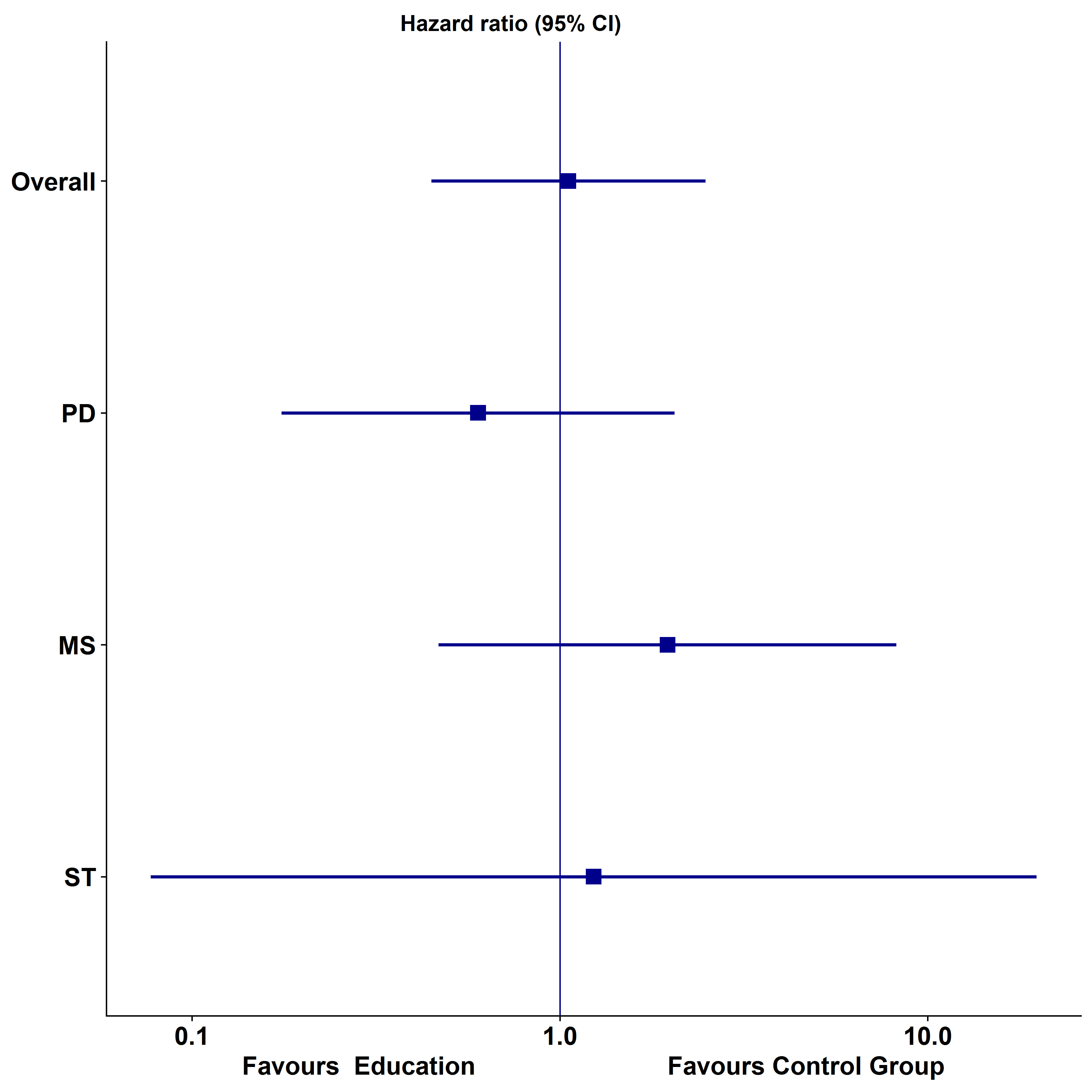

Supplement: Supplementary Figure 1 — Hazard ratios for time to fall for education vs. control group according to subgroups. [file Image_1.jpeg]
